# Antiplatelet Therapy and Anticoagulation before, during, and after Acute Coronary Syndrome

**DOI:** 10.3390/jcm13082313

**Published:** 2024-04-17

**Authors:** Christoph C. Kaufmann, Marie Muthspiel, Laura Lunzer, Edita Pogran, David Zweiker, Achim Leo Burger, Johann Wojta, Kurt Huber

**Affiliations:** 1 3rd Medical Department with Cardiology and Intensive Care Medicine, Klinik Ottakring (Wilhelminenhospital), A-1160 Vienna, Austria; marie_muthspiel@hotmail.com (M.M.); llunzer@gmx.at (L.L.); edita.pogran@gmail.com (E.P.); davidzweiker@gmail.com (D.Z.); achim.leo.burger@gmail.com (A.L.B.); kurt.huber@meduniwien.ac.at (K.H.); 2 Faculty of Medicine, Sigmund Freud University, A-1020 Vienna, Austria; 3 Ludwig Boltzmann Institute for Cardiovascular Research, A-1090 Vienna, Austria; johann.wojta@meduniwien.ac.at; 4 Core Facilities, Medical University of Vienna, A-1090 Vienna, Austria; 5 Department of Internal Medicine II, Division of Cardiology, Medical University of Vienna, Waehringer Guertel 18-20, A-1090 Vienna, Austria

**Keywords:** acute coronary syndrome, myocardial infarction, antiplatelet therapy, anticoagulation, antithrombotic therapy

## Abstract

Acute coronary syndrome (ACS) remains a major challenge in clinical practice, requiring rapid and effective antithrombotic treatment to mitigate adverse ischemic events while minimizing the risk of bleeding. In recent years, results from several clinical trials addressing this issue through various approaches have substantially improved the treatment landscape for patients presenting with ACS. The emergence of new, potent P2Y_12_ inhibitors has significantly enhanced thrombotic risk reduction and different strategies for de-escalating and shortening dual antiplatelet therapy (DAPT) have demonstrated promising outcomes in reducing bleeding rates. Furthermore, data from ongoing trials focusing on novel therapeutic agents and investigating alternative treatment strategies to optimize outcomes for ACS patients are expected in the next few years. In this review, we summarize the current knowledge and emphasize the critical role of individualized treatment approaches tailored to patient-specific risk factors and individual clinical scenarios.

## 1. Acute Antithrombotic Therapy in Acute Coronary Syndrome

### 1.1. Choice of Antiplatelet Therapy

Antiplatelet therapy is an important cornerstone in the treatment of patients with acute coronary syndrome (ACS), proven to improve both the short- and long-term outcomes of this highly vulnerable patient population. The treatment landscape is evolving steadily with the emergence of novel agents with increased efficacy. Therefore, balancing the individual ischemic and bleeding risks on a case by case basis (personalized strategy) is essential in the management of patients with ACS [1].

The 2023 European Society of Cardiology (ESC) Guidelines for the management of ACS recommend timely aspirin treatment, a cyclooxygenase inhibitor, for all patients with a diagnosis of ACS once contraindications for antithrombotic therapy have been ruled out (Class I, A) [2]. A loading dose of aspirin (150–300 mg p.o. or 75–250 mg i.v.) should be given, followed by a low-dose aspirin maintenance dose (75–100 mg p.o.). In addition to aspirin, a P2Y_12_ inhibitor is recommend in all patients with ACS, with ticagrelor and prasugrel being the preferred agents, as they were tested in the randomized controlled PLATO and TRITON TIMI-38 trials [3,4]. For ticagrelor, a loading dose of 180 mg should be given, followed by a maintenance dose of 90 mg twice daily, while for prasugrel, a loading dose of 60 mg is recommended, followed by a maintenance dose of 10 mg once daily. Prasugrel treatment is contraindicated in patients with a history of stroke, and dose adjustments are necessary in those with a body weight of <60 kg and age of ≥75 years [5].

Based on the results of the ISAR-REACT 5 trial—the first trial to directly compare prasugrel and ticagrelor—prasugrel might be considered preferable to ticagrelor in patients with ACS proceeding to primary coronary intervention (PCI) (Class IIa, B). This recommendation is based on the results of the study, which showed significant differences in the primary composite endpoints of death, myocardial infarction, and stroke at 1 year in patients after ACS (9.3% in ticagrelor group vs. 6.9% in the prasugrel group, hazard ratio [HR], 1.36; 95% confidence interval [CI], 1.09 to 1.70; *p* = 0.006). Furthermore, no significant difference in bleeding events was observed between the two P2Y_12_ inhibitors [5]. However, the clinical importance of using prasugrel over ticagrelor in clinical routine is still not widely accepted and is a matter of intense debate [6].

**Figure 1:**
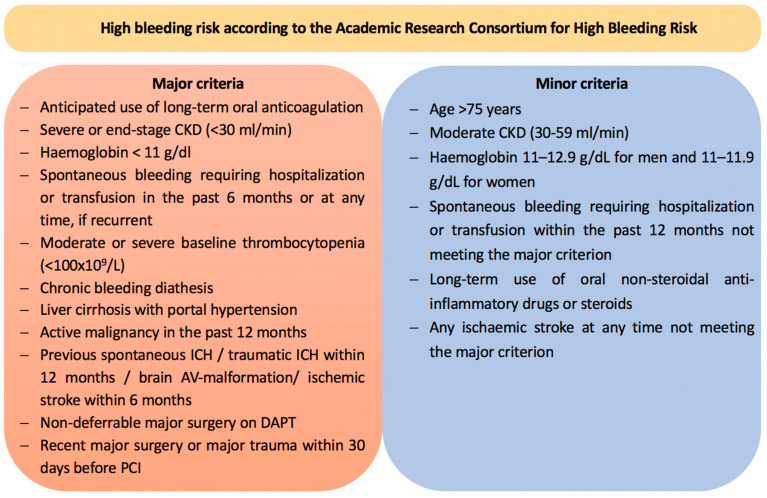
Bleeding risk criteria according to the Academic Research Consortium for High Bleeding Risk, adapted from [2]. AV-malformation = arteriovenous malformation, CKD = chronic kidney disease, and ICH = intracerebral hemorrhage.

In older patients (≥70 years of age) or patients with an increased risk of bleeding, clopidogrel may be considered as the choice of P2Y_12_ inhibitor (Class IIb, B). Bleeding risk can be quantified using the Academic Research Consortium—high bleeding risk (ARC-HBR) criteria, with ≥1 major or ≥2 minor criteria being considered as a high bleeding risk (Figure 1). Since both prasugrel and ticagrelor have a superior efficacy, however, this approach should be reserved for selected patients after critical appraisal. For clopidogrel, a loading dose of 600 mg should be given orally, followed by a maintenance dose of 75 mg once daily in the context of ACS.

### 1.2. Pre-Treatment with P2Y_12_ Inhibitors

While the efficacy and safety of P2Y_12_ inhibitors in the treatment of ACS, in general, are well established, the correct timing of treatment initiation remains a controversial topic with different recommendations for STEMI and NSTE-ACS. An important limitation of pre-treatment with P2Y_12_-ihibitors lies in the potential for the misdiagnosis of ACS [6].

In patients with a working diagnosis of NSTE-ACS, in whom coronary anatomy is not known and early invasive management (<24 h) is planned, routine pre-treatment with a P2Y_12_ inhibitor is not recommended (Class III, A). However, if a delayed invasive strategy (>24 h) is planned, pre-treatment with a P2Y_12_ inhibitor may be considered (Class IIb, C). These recommendations are largely based on the following trials. The ACCOAST trial enrolled 4033 patients with NSTE-ACS scheduled for coronary angiography within 48 h, and randomly assigned them to pre-treatment with prasugrel (30 mg p.o.) or placebo (Table 1). If PCI was considered necessary, an additional 30 mg of prasugrel was given to the pre-treatment group and a full loading of 60 mg was administered to the control group. The primary composite efficacy endpoints (death from cardiovascular causes, myocardial infarction, stroke, urgent revascularization, or glycoprotein IIb/IIIa inhibitor rescue therapy) were similar between the two groups (HR 1.02; 95% CI, 0.84 to 1.25; *p* = 0.81). However, the risk of major bleeding at day 7 was significantly increased in the pre-treatment group (HR 1.90; 95% CI, 1.19 to 3.02; *p* = 0.006) [7]. The DUBIUS trial randomized 1449 patients with NSTE-ACS scheduled for coronary angiography within 72 h to a pre-treatment group with ticagrelor or a no pre-treatment group. Based on prespecified criteria, the trial was stopped by the steering committee due to the risk of futility, as it was deemed highly unlikely that a superiority of either strategy could be found. There was no statistically significant difference between the two groups with regard to the primary efficacy endpoint or the safety endpoint of bleeding events [8]. In the above-mentioned ISAR-REACT 5 trial, patients randomized to ticagrelor had routine pre-treatment, while patients in the prasugrel arm had a deferred loading strategy. One could argue that pre-treatment may have contributed to the observed superiority of prasugrel in this patient population [5]. A meta-analysis pooling data from randomized controlled trials and observational studies investigated the efficacy of pre-treatment with clopidogrel in patients undergoing PCI. Pre-treatment with clopidogrel was not associated with a significant benefit on hard outcomes, especially in the subgroup of patients with NSTE-ACS [9].

In patients undergoing the primary PCI strategy—the optimal guideline-recommended treatment strategy in STEMI—pre-treatment with a P2Y_12_ inhibitor may be considered (Class IIb, B). Although most international STEMI networks advocate routine upstream treatment with P2Y_12_ inhibitors once a working diagnosis of STEMI has been made, this has only been tested for ticagrelor in a randomized controlled fashion in one trial. The ATLANTIC trial randomized 1862 patients with STEMI to a prehospital ticagrelor treatment group or in-hospital (in the catherization lab) treatment with ticagrelor group. A relatively short median time from randomization to angiography of 48 min was reported in the trial, with an even shorter median time difference of 31 min between the two treatment strategies. While there was no significant difference in the rates of major adverse cardiovascular events between the two study groups, the secondary endpoint of early stent-thrombosis was significantly decreased in the pre-treatment group (Odds ratio [OR] 0.19; 95% CI, 0.04 to 0.86; *p* = 0.02). Bleeding complications were low in both study groups, without statistically significant differences [10]. The national SWEDEHEART registry evaluated the efficacy of ticagrelor pre-treatment in 7433 patients in a real-word setting and also found no statistically significant differences in the primary endpoints of 30 day all-cause mortality, myocardial infarction, or stent thrombosis after extensive correction for differences in baseline characteristics. The registry found no increase in major bleeding rates with ticagrelor pre-treatment [24]. Rohla et al. compared the outcomes of 1963 STEMI patients receiving immediate (at time of diagnosis) or delayed (after coronary anatomy) P2Y_12_ inhibitor treatment, using data from the Bern-PCI registry. The authors found similar rates of cardiovascular/cerebrovascular events between patients receiving vs. not receiving pre-treatment (7.1% vs. 8.4%; HR 1.17; 95% CI, 0.78–1.74; *p* = 0.45) [25]. The currently recruiting SOS-AMI trial may shed more light on the benefits and risks of pre-treatment in ASC by assessing the efficacy of self-administered selatogrel, a new P2Y_12_ inhibitor, upon the occurrence of symptoms suggestive of ACS in patients with a history of recent ACS [26].

### 1.3. Anticoagulant Therapy in the Acute Setting (Figure 2)

Next to antiplatelet therapy, parenteral anticoagulation is an important pillar in the treatment of patients with ACS. As such, the current guidelines recommend parenteral anticoagulation for all patients with ACS at the time of diagnosis (Class I, A). Recent data from a meta-analysis found a significantly lower risk of mortality and in-hospital cardiogenic shock, as well as improved reperfusion of the infarct-related artery, with an upstream anticoagulation strategy in patients with STEMI [27]. In patients undergoing PCI, an unfractionated heparin bolus of 70–100 U/kg should be administered as the default strategy, followed by a titrated dosage in the cath-lab to achieve a sufficient activated clotting time (Class I, C).

**Figure 2:**
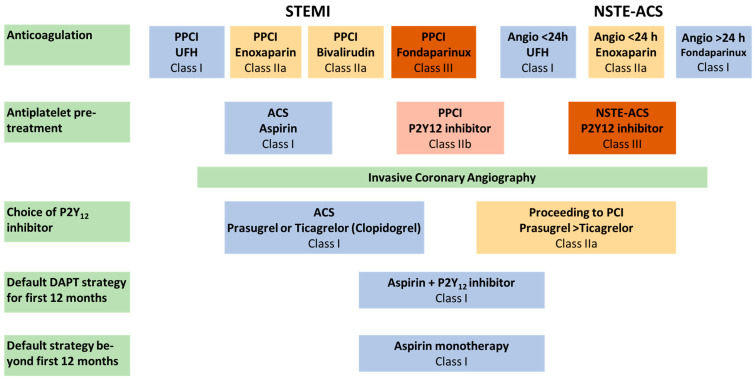
Recommendations for anticoagulation in ACS, adapted from [2] Angio = angiography.

According to the findings of the ATOLL trial, enoxaparin, a low-molecular-weight heparin, should be used as an alternative to unfractionated heparin in STEMI patients (Class IIa, B). This study reported a significant reduction in the primary composite endpoints of death, myocardial infraction complications, procedure failure, and major bleeding at 30 days [28]. Similarly, bivalirudin, a direct thrombin inhibitor, should be used as an alternative to unfractionated heparin (Class IIa, B), based on the findings of the BRIGHT-4 trial, which randomized 6,016 patients with STEMI undergoing primary PCI to bivalirudin plus a high-dose infusion after PCI or unfractionated heparin. A significant reduction in the primary endpoints (all-cause mortality or major bleeding) at 30 days (3.06% vs. 4.39%; *p* = 0.007), as well as a significant reduction in stent thrombosis (0.37% vs. 1.10%; *p* = 1.10%; *p* = 0.0015), was observed [29]. On the other hand, fondaparinux is not recommended in patients with STEMI undergoing PCI (Class III, B) after the OASIS-6 trial failed to show a benefit of this treatment compared to placebo in patients undergoing primary PCI [30]. If enoxaparin is chosen as the anticoagulant agent, a bolus of 1 mg/kg should be administered subcutaneously—for bivalirudin, a 0.75 mg/kg i.v. bolus should be given, followed by an i.v. infusion of 1.75 mg/kg/h for 4 h after the procedure.

Enoxaparin should also be considered as an alternative to unfractionated heparin in NSTE-ACS patients with early invasive angiography (Class IIa, A). Moreover, for patients with NSTE-ACS without an indication for early invasive work-up within 24 h, fondaparinux should be used (Class I, B). This recommendation is based on the findings of the OASIS-5 trial, which randomized 20,078 patients with NSTE-ACS to fondaparinux (2.5 mg daily) or enoxaparin (1 mg per kilogram of body weight twice daily). The participants were allocated to a study group within 24 h of symptom onset. While the primary combined outcome of death, myocardial infarction, and refract ischemia at day 9 was similar between the two groups, a significantly lower risk of bleeding was observed in the fondaparinux group (HR 0.52; 95% CI, 0.44 to 0.61; *p* < 0.001). Fondaparinux treatment was also associated with a significantly reduced mortality at 30 days and 180 days, making it the treatment of choice in this patient population [31].

### 1.4. Parenteral Antiplatelet Therapy

Routine treatment with a Platelet glycoprotein (GP) IIb/IIIa receptor antagonist is not recommended (Class III, A), and is reserved as a bail-out strategy for patients with evidence of no-reflow or thrombotic complications during PCI (Class IIa, C). As shown in a meta-analysis pooling data from major randomized controlled trials, there is no strong evidence for the beneficial effects of GP IIb/IIIa receptor antagonists in routine clinical practice. While a significant reduction in cardiovascular events was observed in patients with a high risk of thrombotic complications, this benefit has to be weighed against a significant increase in bleeding events [32]. If used as a bail-out strategy or for the treatment of thrombotic complications, eptifibatide should be administered with a double bolus of 180 mcg/kg i.v. (given at 10 min intervals), followed by an infusion of 2.0 mcg/kg/min for up to 18 h. Tirofiban should be administered with a bolus of 25 mcg/kg i.v. over a 3 min duration, followed by an infusion of 0.15 mcg/kg/min for up to 18 h.

Cangrelor is an intravenous P2Y_12_ inhibitor, characterized by rapid, predictable, and strong but reversible platelet inhibition. Its plasma half-life lies between 3 and 6 min, and platelet function returns to normal levels around 30–60 min after the termination of treatment. Cangrelor should be administered as an intravenous bolus of 30 μg per kilogram of body weight, followed by an intravenous infusion of 4 μg per kilogram per minute [2,33]. The use of cangrelor has been tested in several clinical trials before (CHAMPION PCI) and at the time of PCI (CHAMPION PLATFORM and CHAMPION PHOENIX) in patients with ACS and chronic coronary syndrome (CCS). In all of these studies, cangrelor was compared to clopidogrel.

In the CHAMPION PCI trial, including 8716 patients with ACS undergoing PCI, cangrelor was shown to be non-superior to clopidogrel regarding the primary efficiency endpoints (composite of death from any cause, myocardial infarction, or ischemia-driven revascularization at 48 h) (OR 1.05; 95% CI, 0.88 to 1.24; *p* = 0.59). Concerning the safety endpoints, minor bleeding events occurred more often in the cangrelor group (OR 1.19, 95% CI, 17.6% to 15.2%; *p* = 0.003), with major bleeding events showing only a numerical difference between the two treatment groups (OR 1.26; 95% CI, 3.6% to 2.9%; *p* = 0.06) [34]. The CHAMPION PLATFORM trial randomized 5362 ACS patients to cangrelor or placebo treatments, followed by clopidogrel treatment for both groups. While the primary efficacy endpoint occurred at a similar rate compared to the placebo, the key secondary endpoint of stent thrombosis was significantly reduced in patients randomized to the cangrelor treatment (OR: 0.31; 95% CI, 0.11 to 0.85; *p* = 0.02). Major bleeding was more often observed in the cangrelor group, primarily driven by a higher rate of groin hematomas [35].

The CHAMPION PHOENIX trial assigned 11,145 patients undergoing elective or urgent PCI to cangrelor or clopidogrel treatment at the time of PCI—43.9% of patients had a diagnosis of ACS. The primary efficiency endpoints (death, myocardial infarction, ischemia-driven revascularization, or stent thrombosis) were significantly lower among the patients treated with cangrelor when compared to those receiving clopidogrel (4.7% vs. 5.9%, OR 0.78; 95% CI, 0.66% to 0.93%; *p* = 0.005). Regarding the secondary efficacy endpoint, the incidence of stent thrombosis was notably reduced in the cangrelor group when compared to the clopidogrel group (0.8% vs. 1.4%, OR 0.62; 95% CI, 0.43 to 0.90; *p* = 0.01). At 30 days, the rate of the composite efficiency endpoint remained lower in the cangrelor arm (6.0% vs. 7.0%, OR 0.85, 95% CI; 0.73 to 0.99; *p* = 0.03). The number of severe bleeding events was low in both groups, with a statistically higher rate of ACUITY bleeding events occurring with the cangrelor treatment (4.3 vs. 2.5%; OR 1.72; 95% CI: 1.39 to 2.13; *p* < 0.001) and no difference in GUSTO bleeding events (0.16 vs. 0.11%; OR 1.50; 95% CI, 0.53 to 4.22; *p* = 0.44) [36].

A trial-level meta-analysis pooling data from the three CHAMPION trials showed no significant difference with cangrelor treatment for all-cause mortality (RR 0.72, 95% CI 0.36–1.43) or myocardial infarction (RR 0.94, 95% CI 0.78–1.13). However, cangrelor significantly reduced the risk of ischemia-driven revascularization (RR 0.72, 95% CI 0.52–0.98) and stent thrombosis (RR 0.60, 95% CI 0.44–0.82). There were also no significant differences with regard to GUSTO or TIMI major bleeding events—the more sensitive ACUITY-defined bleeding-criteria was not assessed [37].

Overall, it is notable that data for cangrelor in combination with ticagrelor or prasugrel are quite limited and minor bleeding events are observed more often with cangrelor compared to clopidogrel. In addition, the question persists as to why cangrelor did not consistently demonstrate superiority in the aforementioned trials, since clopidogrel achieves maximum platelet inhibition between only 4 and 6 h following oral administration, whereas an optimal antiplatelet effect can be observed within minutes of intravenous cangrelor injection. This discrepancy may be attributed to variations in study design (for example, the upfront administration of clopidogrel in CHAMPION PCI) and to the shorter duration of cangrelor infusion, although the latter can be avoided by a prolonged administration of 4 h. Moreover, achieving optimal blood flow during the early stages of the procedure may be of even more importance than optimal platelet inhibition. In conclusion, cangrelor may only be considered on a case-by-case basis in selected P2Y_12_-receptor-naive patients undergoing PCI, including patients who are not suitable for oral drug therapy, because of its proven effects on preventing intra- and post-procedural stent thrombosis (Class IIb, Level A).

## 2. Long-Term Antithrombotic Therapy in Acute Coronary Syndrome

### 2.1. Shortening the Duration of Dual Antiplatelet Therapy

Post-interventional dual antiplatelet therapy (DAPT) is the cornerstone in the prevention of thrombo-ischemic events in patients with ACS. In general, standard treatment consisting of aspirin and a potent P2Y_12_ inhibitor (ticagrelor or prasugrel) for 12 months followed by lifelong aspirin monotherapy is recommended for all patients after PCI (Class I, A). Alternatively, long-term P2Y_12_ inhibitor monotherapy can be considered instead of aspirin (Class IIb, A) [2]. It is widely recognized that the antithrombotic efficacy of DAPT is greatest in the acute and early stages after the index event, while bleeding risk remains high throughout therapy [3]. In this respect, novel antithrombotic treatment strategies, such as shortening DAPT followed by P2Y_12_ inhibitor monotherapy or the de-escalation of DAPT from potent P2Y_12_ inhibitors to clopidogrel, have been recently investigated in several clinical trials in order to reduce bleeding rates without increasing ischemic risk.

The TWILIGHT trial, including 7119 patients after PCI (64% ACS, 36% CCS), investigated a strategy of abbreviated DAPT followed by P2Y_12_ inhibitor monotherapy [12]. Ticagrelor monotherapy after 3 months of DAPT was associated with a 44% lower risk of bleeding (BARC type 2, 3, or 5) when compared to standard DAPT (HR 0.56; 95% CI, 0.45 to 0.68; *p* < 0.001), with no significant increase in MACE (death, MI, stroke) (HR 0.99; 95% CI, 0.78 to 1.25; *P*_non-inferiority_ < 0.001) at 15 months [11]. The limitations of this study included that the patients had a low baseline risk of bleeding and the inclusion criteria for ACS were confined to NSTE-ACS. Nevertheless, several prespecified subgroup-analyses, including high-bleeding-risk (HBR) patients, demonstrated consistent outcomes [38,39,40,41,42]. A similar but even shorter DAPT regimen (1 month of DAPT followed by 23 months of ticagrelor monotherapy) was investigated in low-bleeding-risk patients in the GLOBAL LEADERS trial. While the results did not demonstrate a significant association with fewer primary endpoint events in the short DAPT group (all-cause death, MI) (RR 0.87; 95% CI 0.75 to 1.01; *p* = 0.073), non-inferiority to standard therapy was met. Additionally, the bleeding rates (BARC type 3, or 5) were similar between groups (2.04% vs. 2.12%, RR 0.97; 95% CI 0.78 to 1.20; *p* = 0.77 [43].

The TICO trial was the only study to examine the effect of early ticagrelor monotherapy exclusively in ACS (36% STEMI) patients, showing a reduction in primary adverse clinical events (TIMI major bleeding, all-cause death, myocardial infarction [MI], stent thrombosis [ST], stroke, and target vessel revascularization) (HR 0.66; 95% CI, 0.48 to 0.92; *p* = 0.01) and major bleeding (HR 0.56; 95% CI, 0.34 to 0.91; *p* = 0.02) when switching to ticagrelor monotherapy after 3 months of DAPT compared to the default strategy [12].

**Figure 3:**
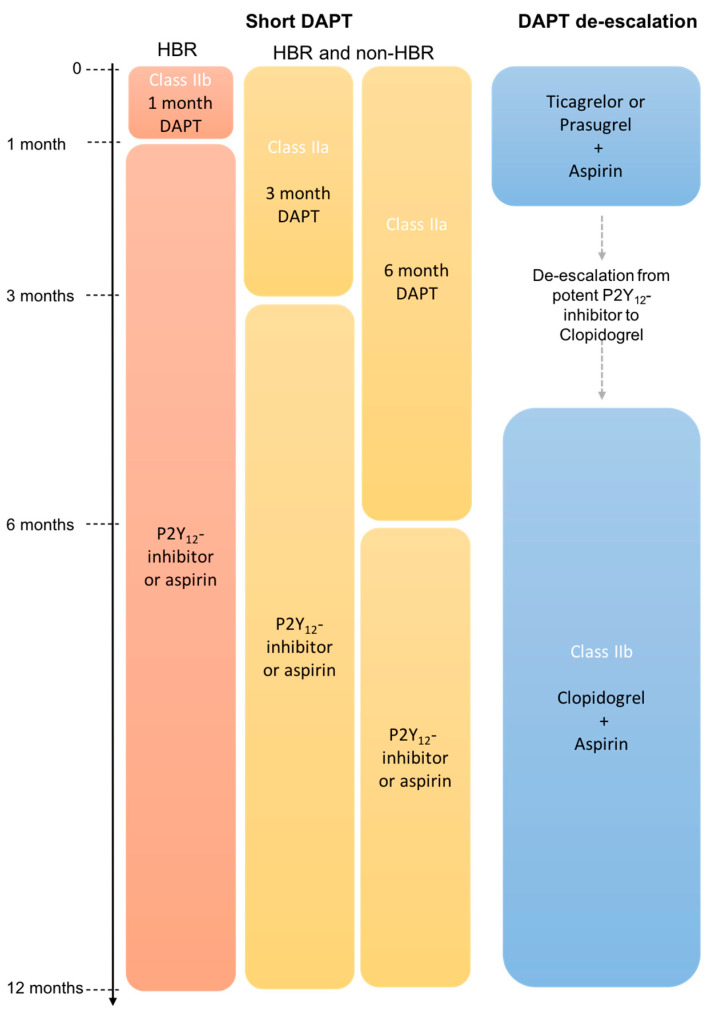
Recommendations for shortening/de-escalation of DAPT, adapted from [2] HBR = high bleeding risk.

One-year data on clopidogrel monotherapy following abbreviated DAPT were provided by the following trials. While the STOPDAPT-2 trial showed reduced bleeding rates (TIMI major or minor bleeding) for very early clopidogrel monotherapy (1 month) in predominantly stable patients (ACS 38%, CCS 62%), the results from the STOPDAPT2-ACS trial, failing to meet its primary non-inferiority endpoints (of CV death, MI, ST, stroke, or TIMI major or minor bleeding) at 12 months (HR 1.14; 95% CI, 0.80 to 1.6; *P*_non-inferiority_ = 0.06), indicated that this does not apply to patients in the acute setting [13,14]. In both trials, only patients with a low to intermediate bleeding risk were included. In this regard, the results of the MASTER-DAPT trial, being the first to selectively enroll HBR patients (ACS 49%, CCS 51%), demonstrated that, even in this cohort, an abbreviated DAPT regimen followed by clopidogrel monotherapy presents a safe strategy for preventing bleeding events after PCI. [15] In detail, 1 month of DAPT proved non-inferior to standard therapy in terms of the primary combined endpoint (all-cause death, MI, stroke, and BARC type 3 or 5) (−0.23 percentage points; 95% CI, −1.80 to 1.33; *P*_non-inferiority_ < 0.001), while being associated with lower bleeding rates (BARC type 2, 3, or 5) (6.5% vs. 9.11%, 95% CI, −4.40 to −1.24, *P*_non-inferiority_ < 0.001) at 11 months [15]. Based on the aforementioned trials, single antiplatelet therapy (preferably with a P2Y_12_ inhibitor) should be considered in patients without a high ischemic risk and without ischemic events after 3 to 6 months of DAPT (Class IIa, A) (Figure 3). In HBR patients, aspirin or P2Y_12_ inhibitor monotherapy after 1 month of DAPT may be considered (Class IIb, B). In view of data from multiple randomized controlled trials demonstrating safety benefits in terms of bleeding rates by shortening DAPT, one might question that the recent guidelines recommend the abbreviated regimen only as an alternative strategy to default therapy. In this respect, the studies to date exhibit important limitations, some of which have already been mentioned. Besides being performed on relatively selectED populations (mainly low to medium bleeding risk, underrepresentation of ACS), the trials presented a one-year non-inferiority design and were primarily powered for bleeding endpoints. Several ongoing trials are addressing these issues through various approaches. The South Korean A-CLOSE trial is investigating prolonged clopidogrel-based DAPT versus clopidogrel monotherapy from 12 months after PCI in patients at a high risk for either bleeding or ischemic complications. Extended clopidogrel-based DAPT is further being currently compared to clopidogrel or ticagrelor monotherapy at 36 months after PCI in the SMART-CHOICE II trial. Regarding the optimal choice of agent for monotherapy, the SMART-CHOICE III trial randomizes patients at 12 months after PCI to either aspirin or clopidogrel monotherapy. In addition, 5 year data on aspirin versus clopidogrel monotherapy are expected from the STOPDAPT-2 trial in the next few years.

### 2.2. De-Escalation of Dual Antiplatelet Therapy

Switching from a potent P2Y_12_ inhibitor—ticagrelor or prasugrel—to clopidogrel presents another therapeutic option to reduce bleeding risk with DAPT after ACS. So-called de-escalation may be considered as an alternative DAPT strategy (Class IIb, A), but is not recommended within 30 days after the event (Class III, A) (Figure 3). The present recommendation resulted from the following trials. The TROPICAL-ACS trial investigated a platelet function test—a guided switch to aspirin and clopidogrel in STEMI and NSTE-ACS patients after 2 weeks of standard DAPT with ticagrelor or prasugrel [16]. The de-escalation regimen demonstrated non-inferiority compared to the default strategy in terms of the primary combined endpoint (CV death, MI, stroke, and BARC type ≥ 2) (HR 0.81; 95% CI, 0.62 to 1.06; *P*_non-inferiority_ = 0.0004) at 12 months after PCI [16]. The POPULAR GENETICS trial randomized 2499 STEMI patients to clopidogrel-based DAPT or the continuation of default therapy within 3 days after PCI [17]. At 12 months, a genetic-testing-guided de-escalation to clopidogrel was non-inferior with respect to net clinical events (all-cause death, MI, ST, stroke, and Platelet Inhibition and Patient Outcomes (PLATO) major bleeding) (95% CI, 2.0 to 0.7; *P*_non-inferiority_ < 0.001) and resulted in lower bleeding rates (HR 0.78; 95% CI, 0.61 to 0.98; *p* = 0.04) when compared to standard DAPT [17]. Consistent results were provided by the monocentric TOPIC trial, showing the superiority of a de-escalation approach 30 days after PCI in terms of the primary combined endpoint of CV death, urgent revascularization, stroke, and bleeding events (HR 0.48; 95% CI 0.34–0.68; *p* < 0.01) [18]. In the East Asian population, the TALOS-AMI trial demonstrated in 2697 patients that switching to clopidogrel-based DAPT 30 days after PCI reduced net clinical events (CV death, MI, stroke, and BARC type 2, 3, and 5) (HR 0.55; 95% CI, 0.40 to 0.76; *P*_non-inferiority_ < 0.001) and bleeding rates (BARC type 2, 3, or 5) (HR 0.52; 95% CI, 0.35 to 0.77; *p* = 0.0012) one year after the index event [19]. The above-mentioned trials were, thus, able to show that a de-escalation approach was associated with an overall decrease in bleeding events, but did not increase the risk of ischemia. However, it is essential to interpret these findings within the context of certain limitations, as long-term data (>12 months) and randomized controlled trials primarily powered for ischemic endpoints are lacking. Additionally, the question of which patient populations the above results and the current recommendations are suitable for arises, as patients at a high thrombotic risk were underrepresented.

When employing clopidogrel, interpatient genetic variability in terms of pharmacological response should always be taken into account [44]. Genotype or platelet function testing to identify patients at a high ischemic risk was used in the POPULAR-GENETICS and TROPICAL-ACS trials, respectively, whereas unguided de-escalation was performed in the TALOS-AMI and TOPIC trials [16,17,18,19]. Compared to the previous 2019 version, the new ACS guidelines no longer mention a genotype- or platelet-function-guided approach prior to a de-escalation strategy in ACS patients to prevent potential ischemic events [2].

### 2.3. Prolonging Dual Antiplatelet Therapy

In specific scenarios with an increased ischemic risk, the extension of DAPT beyond 12 months may be necessary for long-term secondary prevention. According to guidelines, in patients with a high thrombotic risk (HTR) and without HBR, extended long-term treatment with a second antithrombotic agent in addition to aspirin should be considered (Class IIa, A). In patients with a moderate ischemic risk, extended long-term treatment remains a Class IIb recommendation (Level A) [2]. Classification for HTR encompasses complex coronary artery disease (CAD) and at least one of the following risk factors: diabetes mellitus requiring medication, recurrent MI, polyvascular disease, chronic kidney disease (CKD), multivessel, premature (<45 years) or accelerated (new lesion within 2 years) CAD, and concomitant systemic inflammatory disease. Patients classified as having a moderate thrombotic risk exhibit non-complex CAD and at least one of the first four factors mentioned. The current recommendations are based on the results of the PEGASUS-TIMI 54 trial, showing a significant risk reduction in MACE for an extended ticagrelor-based DAPT (60 mg ticagrelor BID) regimen in ACS patients with at least one ischemic risk factor when compared to aspirin monotherapy after 12 months of DAPT (HR 0.84; 95% CI, 0.74 to 0.95, *p* = 0.004) [45]. A dose reduction to 60 mg b.i.d. should be considered, as low-dose ticagrelor was associated with fewer bleeding complications than a maintenance dose of 90 mg b.i.d. [2]. Prolonged clopidogrel- and prasugrel-based DAPT were investigated in the DAPT trial and shown to reduce the risk of stent thrombosis (HR 0.29; 95% CI 0.17 to 0.48; *p* < 0.001), as well as major adverse cardiovascular and cerebrovascular events (HR 0.71; 95% CI 0.59 to 0.85; *p* < 0.001). Notably, bleeding rates were significantly increased when compared to aspirin monotherapy (2.5% vs. 1.6%, *p* = 0.001), underscoring the limitation of current recommendations for patients who do not exhibit HBR [46].

### 2.4. ACS Patients with an Indication for Long-Term Anticoagulation

**Figure 4:**
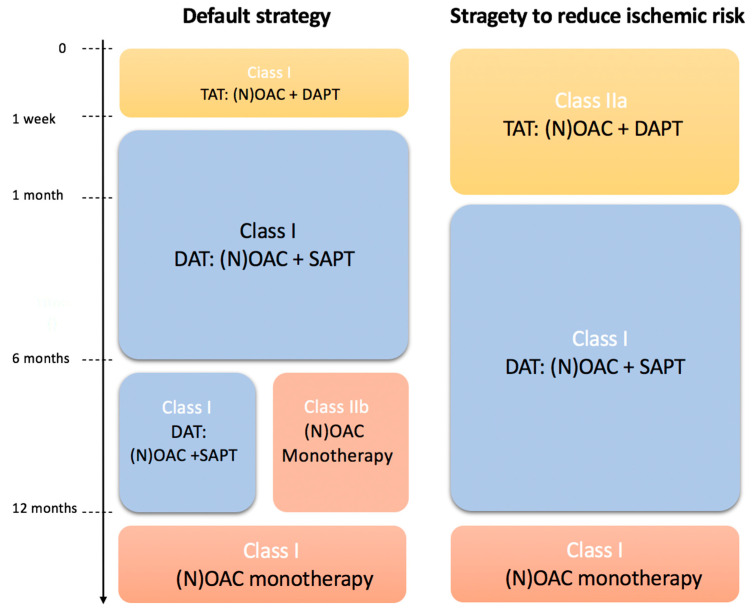
Recommendations for TAT/DAT, adapted from [2] DAT = dual antithrombotic therapy, TAT = triple antithrombotic therapy.

A considerable proportion of patients presenting with ACS have an indication for long-term anticoagulation (6–8%). These patients are at an increased risk of bleeding and require careful management tailored to their individual risk profile. The default strategy for patients with ACS and a long-term indication for anticoagulation involves antithrombotic triple therapy (TAT) for up to one week (NOAC + aspirin + clopidogrel), followed by dual antithrombotic therapy (DAT) for up to 12 months (NOAC + clopidogrel or aspirin) (Class I, A). In patients at a high ischemic risk or with anatomical/procedural features that are assumed to outweigh the bleeding risk, TAT for longer than one week and up to one month should be considered (Class IIa, C) (Figure 4). The use of ticagrelor or prasugrel as part of TAT is not recommended, as neither have been sufficiently tested in randomized controlled trials, based on their increased risk of bleeding compared to clopidogrel (Class III, C) [2]. Recommendations for patients with long-term anticoagulation are derived from four large randomized controlled trials (RE-DUAL-PCI, PIONEER-AF-PCI, AUGUSTUS, and ENTRUST AF-PCI). All of these trials were powered to test the safety of these substances with regard to bleeding events, but not to assess differences in ischemic events [20,21,22,23].

RE-DUAL-PCI included a total of 2725 patients with atrial fibrillation who had undergone PCI, with 50.5% of patients having ACS. The patients were randomized to either TAT with warfarin, a P2Y_12_ inhibitor (clopidogrel or ticagrelor), and aspirin (for 1 to 3 months) or DAT including dabigatran (110 mg or 150 mg twice daily) plus a P2Y_12_ inhibitor (clopidogrel or ticagrelor). The primary endpoint of major or non-major bleeding events occurred significantly less frequently in the DAT vs. the TAT group, with 15.4% in the 110 mg dual-therapy group vs. 26.9% in the triple-therapy group (HR 0.52; 95% CI, 0.42 to 0.63; *P*_non-inferiority_ < 0.001) and 20.2% in the 150 mg dual-therapy group vs. 25.7% in the corresponding triple-therapy group (HR 72; 95% CI, 0.58 to 0.88; *P*_non-inferiority_ < 0.001). While the efficacy, defined as a composite of thromboembolic events, death, or unplanned revascularization, of DAT was non-inferior to TAT (HR 1.04; 95% CI, 0.84 to 1.29; *P*_non-inferiority_ = 0.005), a significantly higher risk of stent thrombosis was observed in the DAT group treated with 110 mg of dabigatran (twice as often compared to TAT). However, it is important to note that the trial was underpowered for the detection of individual ischemic endpoints [21].

The PIONEER AF-PCI trial enrolled a total of 2124 patients with atrial fibrillation who had undergone PCI (52.3% ACS). Within 72 h after sheath removal, the patients were assigned to one of the following three arms: DAT with rivaroxaban at 15 mg once daily plus a P2Y_12_ receptor inhibitor for 12 months (group 1), experimental TAT with rivaroxaban at 2.5 mg twice daily plus DAPT for 1, 6, or 12 months (group 2), or standard TAT with VKA plus DAPT for 1, 6, or 12 months (group 3). The primary safety endpoint, defined as major or minor bleeding events, was most commonly observed in group 3 (26.7%), followed by 18% in group 2 and 16.8% in group 1 (HR for group 1 vs. group 3, 0.59, 95% CI, 0.47 to 0.76, *p* < 0.001; HR for group 2 vs. group 3, 0.63, 95% CI, 0.50 to 0.80, *p* < 0.001). A subgroup analysis in patients with ACS revealed a tendency towards clinically significant bleeding events in group 2 when compared to group 1. Rates for the composite cardiovascular endpoints (death from cardiovascular causes, myocardial infarction, stroke, and stent thrombosis) did not differ significantly (6.5% of the patients in group 1, 5.6% of the patients in group 2, and 6% of patients in group 3 (*p* > 0.05)) [20].

In the AUGUSTUS trial, DAT consisting of apixaban and a P2Y_12_ inhibitor was investigated in comparison to a combination of VKA and a P2Y_12_ inhibitor or TAT (in addition to aspirin) in 4614 patients with atrial fibrillation who had recent ACS (37.3%) or PCI. Regarding the primary outcome of major or clinically non-major bleeding, a relative risk reduction of 31% was observed with NOAC when compared to VKA (HR 0.69, 95% CI, 0.58 to 0.81, *P*_non-inferiority and superiority_ < 0.001). Additional treatment with aspirin led to a significant higher risk of major or clinically non-major bleeding (HR 1.89; 95% CI, 1.59 to 2.24; *p* < 0.001). Further, death or hospitalization occurred less in patients treated with apixaban than in those receiving VKA (23.5% vs. 27.4%, HR 0.83, 95% CI, 0.74 to 0.93, *p* = 0.002). Overall, there was no difference observed regarding ischemic events [22].

ENTRUST-AF PCI included a total number of 1506 patients with atrial fibrillation after successful PCI (50% ACS). The patients were randomly assigned to TAT with full-dose edoxaban or VKA. The results demonstrated the non-inferiority of edoxaban in terms of bleeding rates when compared to VKA (95% CI, 0.65–1.05; *P*_non-inferiority_ = 0.001, HR 1.20; *P*_superiority_ = 0.1154). While there was no significant difference regarding ischemic events, a numerical trend towards a higher number of early ischemic events was observed between the two groups [23].

A meta-analysis including data from the four aforementioned trials confirmed a significant risk reduction in the primary endpoint of major or clinically non-major bleeding with DAT when compared to TAT (RR 0.66; 95% CI, 0.56–0.78; *p* < 0.001), with all trials meeting the criteria of non-inferiority. Patients receiving DAT were not at a higher risk for major cardiovascular events (all-cause death, stroke, or trial-defined MACE), however, a numerically higher risk of MI (RR 1.22, 95% CI, 0.99–1.52; *p* = 0.07) and a significantly higher risk of stent thrombosis (RR 1.59, 95% CI, 1.01–2.50; *p* = 0.04) were reported in patients with DAT compared to patients with TAT. When interpreting the results of these studies, it is notable that the study results were confounded by the use of NOACs in the DAT treatment groups and VKA in the TAT treatment groups in all trials, except for AUGUSTUS. Additionally, all patients randomized to DAT had a short-term period with aspirin, effectively creating a window of TAT immediately after PCI [47].

If a VKA is the anticoagulant of choice in the context of TAT/DAT, a target INR of 2–2.5 is recommended by the current guidelines (Class IIa, B). If rivaroxaban is used and the bleeding risk exceeds the ischemic risk, a reduced dose of 15 mg per day instead of 20 mg per day during DAT/TAT should be considered (Class IIa, B). Similarly, for dabigatran, a reduced dose of 110 mg per day instead of 150 mg per day is recommended in patients with a high bleeding risk during DAT/TAT. For apixaban and edoxaban, full-dose anticoagulation should be used, irrespective of the duration of DAT/TAT, and dose reduction should be based on the usual criteria of renal function, age, body weight, and concomitant medications [2].

The AFIRE trial included 2236 patients with atrial fibrillation who had undergone PCI or coronary artery bypass grafting more than one year earlier or had stable, angiographically confirmed CAD. The patients were randomized to rivaroxaban monotherapy or DAT with rivaroxaban and an antiplatelet agent. Rivaroxaban monotherapy was noninferior to DAT with regard to efficacy and superior with regard to the safety endpoint, defined as major bleeding in patients with atrial fibrillation and stable coronary artery disease. These findings affirm the current recommendations by international guidelines advocating for the cessation of antiplatelet agents in patients on oral anticoagulation one year after PCI. The limitations of the study are the lack of blinding, the early discontinuation, and its execution in Japan with a reduced rivaroxaban dosage (15 mg or 10 mg depending on renal function), which limits its generalizability for non-Asian patients [48]. In general, the identification of patients with an increased risk of ischemic events and no indication for anticoagulation remains important in order to take preventive measures at an early stage. Available data have shown that the CHA2DS2-VASC Score can be useful to stratify these patients concerning their long-term prognosis, irrespective of the presence of atrial fibrillation [49,50,51].

## 3. Conclusions

Antithrombotic treatment remains a key feature in the medical management of patients with ACS, yielding a positive net clinical benefit. The introduction of newer, more potent P2Y_12_ inhibitors has improved the treatment of ACS substantially, with selected patients benefiting from intravenous platelet inhibition instead of oral medication in the acute setting. The decision for pre-treatment with P2Y_12_ inhibitors remains a topic of discussion and warrants critical clinical judgement on a case-by-case basis, with different implications in STEMI and NSTE-ACS. Recently, several strategies have been proposed to reduce bleeding events in patients with ACS. These strategies encompass the early cessation of DAPT (1-3-6 months after the index event), transitioning from potent P2Y_12_ inhibitors (ticagrelor or prasugrel) to the less potent clopidogrel, or a dose reduction of prasugrel (de-escalation strategy) no earlier than 1 month after the index event. While a brief period of TAT during the index hospitalization should be employed for patients with ACS and an indication for long-term anticoagulation (1 week or up to 1 month), DAT has emerged as the default strategy thereafter, but should not exceed 12 months.

## Figures and Tables

**Table 1:** Selected randomized controlled trials on pre-treatment in ACS, shortening of DAPT, de-escalation of DAPT and Triple- and Dual antithrombotic therapy. ARR: absolute risk reduction; BARC: Bleeding Academic Research Consortium; CI: confidence interval; CRNM: clinically relevant non-major bleeding; CV: cardiovascular; DAPT: dual antiplatelet therapy; DAT: dual antithrombotic therapy; HR: hazard ratio; IRA: infarct related artery; ISTH: International Society for Thrombosis and Hemostasis; MI: Myocardial infarction; mo: months; NSTE-ACS: Non-ST elevation-acute coronary syndrome; PCI: percutaneous coronary intervention; PLATO: Platelet Inhibition and Patient Outcomes; pts: patients; ST: stent thrombosis; STEMI: ST-elevation myocardial infarction; TAT: triple antithrombotic therapy; TIMI: Thrombolysis in Myocardial Infarction; TVR: target vessel revascularization.

Study		Year	Study Population	Study Design	Follow-Up	Primary-Endpoint	Results
**PRE-TREATMENT IN ACS**							
	**ACCOAST [7]**	2013	4033 pts	Pre-treatment with prasugrel vs. placebo (NSTE-ACS)	30 days	death from CV causes, MI, stroke, urgent revascularization or GP IIb/IIIa bailout	HR 1.02; 95% CI, 0.84 to 1.25; *p* = 0.81
	**DUBIUS [8]**	2020	1499 pts	Pre-treatment with ticagrelor vs. no pre-treatment (NSTE-ACS)	30 days	death due to vascular causes, non-fatal MI or non-fatal stroke	ARR: –0.46; 95% CI: –2.87 to 1.89
	**ATLANTIC [10]**	2014	1862 pts	Pre-treatment with ticagrelor vs. in-hospital treatment with ticagrelor (STEMI)	30 days	70% or greater resolution of ST-elevation / no TIMI flow grade 3 in the IRA	ST-elevation: OR 0.93; 95% CI, 0.69 to 1.25; *p* = 0.63 TIMI flow: 0.97; 95% CI, 0.75 to 1.25; *p* = 0.82
**SHORTENING OF DAPT**							
	**TWILIGHT [11]**	2019	7119 pts	3 vs. 12 mo ticagrelor-based DAPT	15 mo	BARC type 2,3, or 5	HR 0.99; 95% CI, 0.78 to 1.25; *P*_non-inferiority_ < 0.001
	**TICO [12]**	2020	3056 pts	3 vs. 12 mo ticagrelor-based DAPT	12 mo	TIMI major bleeding, all-cause death, MI, ST, stroke, or TVR	HR 0.66; 95% CI, 0.48 to 0.92; *p* = 0.01
	**STOPDAPT-2 [13]**	2019	3045 pts	1 vs. 12 mo clopidogrel-based DAPT	12 mo	CV death, MI, ST, stroke, or TIMI major or minor bleeding	HR 0.26, 95% CI, 0.11 to 0.64, *p* = 0.004
	**STOPDAPT2-ACS [14]**	2022	4169 pts	1 vs. 12 mo clopidogrel-based DAPT	12 mo	CV death, MI, ST, stroke, or TIMI major or minor bleeding	HR 1.14; 95% CI, 0.80 to 1.6; *P*_non-inferiority_ = 0.06
	**MASTER-DAPT [15]**	2021	4434 pts	1 vs. ≥ 3 mo clopidogrel-based DAPT	11 mo	all-cause death, MI, stroke, or BARC type 3, or 5	−0.23 percentage points; 95% CI, −1.80 to 1.33; *P*_non-inferiority_ < 0.001
**DE-ESCALATION OF DAPT**							
	**TROPICAL-ACS [16]**	2017	2610 pts	de-escalation to clopidogrel-based DAPT at day 7–14 after discharge vs. standard DAPT	12 mo	CV death, MI, stroke, BARC type ≥ 2	HR 0.81; 95% CI, 0.62 to 1.06; *P*_non-inferiority_ = 0.0004
	**POPULAR GENETICS [17]**	2019	2499 pts	de-escalation to clopidogrel-based DAPT at day 1 to 3 after PCI vs. standard DAPT	12 mo	all-cause death, MI, ST, stroke, or PLATO major bleeding	95% CI, 2.0 to 0.7; *P*_non-inferiority_ < 0.001
	**TOPIC [18]**	2017	646 pts	de-escalation 30 days after PCI to clopidogrel-based DAPT vs. standard DAPT	12 mo	CV death, TVR, stroke, BARC type ≥ 2	HR 0.48; 95% CI 0.34–0.68; *p* < 0.01
	**TALOS-AMI [19]**	2021	2697 pts	de-escalation 30 days after PCI to clopidogrel-based DAPT vs. standard DAPT	12 mo	CV death, MI, stroke, BARC type 2,3, or 5	HR 0.55; 95% CI, 0.40 to 0.76; *P*_non-inferiority_ < 0.001
**TRIPLE- AND DUAL ANTITHROMBOTIC THERAPY**							
	**PIONEER AF-PCI [20]**	2016	2124 pts	DAT with rivaroxaban (gr 1) vs. TAT with low-dose rivaroxaban (gr 2) vs. TAT with VKA (gr 3)	36 mo	TIMI major bleeding or minor bleeding or bleeding requiring medical attention	gr 1 vs. gr 3: HR 0.59; 95% CI, 0.47 to 0.76; *p* < 0.001 gr 2 vs. gr 3: HR 0.63; 95% CI, 0.50 to 0.80; *p* < 0.001
	**RE-DUAL-PCI [21]**	2018	2725 pts	DAT with dabigatran vs. TAT with VKA	14 mo	ISTH major or clinically relevant non major bleeding	110 mg: HR 0.52; 95% CI, 0.42 to 0.63; *P*_superiority_ < 0.001 150 mg: HR 0.72; 95% CI, 0.58 to 0.88; *P*_non-inferiority_ < 0.001
	**AUGUSTUS [22]**	2019	4614 pts	DAT with apixaban vs. DAT with VKA TAT with apixaban vs. TAT with VKA	6 mo	ISTH major or clinically relevant non-major bleeding	Apixaban vs. VKA: HR, 0.69; 95% CI, 0.58 to 0.81; *p* < 0.001 DAT vs. TAT: HR 1.89; 95% CI, 1.59 to 2.24; *p* < 0.001
	**ENTRUST AF-PCI [23]**	2019	1506 pts	DAT with edoxaban vs. TAT with VKA	12 mo	Major or clinically relevant non-major bleeding (CRNM)	HR 0.83; 95% CI 0.65–1.05; *P*_non-inferiority_ = 0.001
